# Exploring cardiovascular risk in subclinical hypothyroidism: The impact of TSHR gene expression and systemic biomarkers

**DOI:** 10.34172/jcvtr.026.33666

**Published:** 2026-03-30

**Authors:** Manjusha Kottola, Desigamani Kanniyappan, Damodaran Boopathi, Jeyapal Vidhyadharan, Vishnu Manjerikattil Govindan, Vineetha Vijayan, Joby Paruthiparayil Jose, Jisha Anjali Madhavan Pillai, Rajitha Puthiya Purayil, Swathi Thoduvayil, Midhun Thazhissery Mohanan, Dinesh Roy Divakaran

**Affiliations:** ^1^Meenakshi Academy of Higher Education and Research (MAHER- Deemed to be University), Chennai, India; ^2^Department of Biochemistry, Madha Medical College and Hospital, Kovur, Chennai, India; ^3^Department of Psychology, Meenakshi Academy of Higher Education and Research (MAHER- Deemed to be University), Chennai, India; ^4^Hridayala Heart and Robotic Research Centre Pvt Ltd, Thiruvananthapuram, India; ^5^Genetika, Centre for Advanced Genetic Studies, Thiruvananthapuram, India

**Keywords:** Subclinical hypothyroidism, TSH receptor gene expression, Cardiovascular risk, Interleukin-6, 3-Nitrotyrosine

## Abstract

**Introduction:**

Subclinical hypothyroidism (SCH), defined by elevated thyroid-stimulating hormone (TSH) with normal thyroid hormone levels, affects approximately 3–8% of the population. Growing evidence indicates that thyroid-stimulating hormone receptor (TSHR) expression in cardiovascular tissues, together with systemic inflammation (IL-6) and oxidative stress (3-nitrotyrosine), may contribute to cardiovascular dysfunction in SCH. This study aimed to evaluate the association between TSHR gene expression and systemic biomarkers—hormonal, inflammatory, and oxidative—and to assess their predictive value for cardiovascular risk in SCH patients.

**Methods:**

A case–control design was adopted involving 150 SCH subjects and 150 age- and sex-matched healthy controls. Demographic and clinical data were collected using structured interviews. Fasting blood samples were analyzed for TSH (CLIA), IL-6, and 3-nitrotyrosine (ELISA), while TSHR gene expression was quantified using real-time PCR with GAPDH as an internal control. Statistical analyses were performed using Stata version 17.0.

**Results:**

Compared with controls, SCH patients showed significantly higher levels of TSH, IL-6, 3-nitrotyrosine, and TSHR gene expression (*P*<0.05). Receiver operating characteristic (ROC) analysis revealed high diagnostic accuracy for these parameters. Although univariate analysis associated TSHR dysregulation with SCH, multivariate regression identified TSH as the only independent predictor of disease status. Oxidative stress demonstrated a significant positive association with TSHR expression, whereas IL-6 showed no independent influence.

**Conclusion:**

SCH is characterized by elevated hormonal, oxidative, and inflammatory markers, with oxidative stress contributing to TSHR upregulation. TSH remains the strongest independent predictor, underscoring the hormonal–oxidative interaction in cardiovascular risk among SCH patients.

## Introduction

 Hypothyroidism is a clinical condition characterized by elevated serum thyroid-stimulating hormone (TSH) levels accompanied by reduced free thyroxine (FT4) concentrations. Free triiodothyronine (FT3) levels may be decreased or may remain within normal limits.^1^ In contrast, subclinical hypothyroidism (SCH), also known as mild thyroid failure, is defined by an increased TSH level while free T4 and T3 levels remain within the normal physiological range. This biochemical pattern is relatively common, affecting an estimated 3% to 8% of individuals in the general population without a prior diagnosis of thyroid dysfunction.^2^ The subtle hormonal imbalance observed in SCH may trigger a cascade of molecular and cellular alterations that extend beyond thyroid hormone levels, potentially contributing to increased cardiovascular vulnerability.

 While conventional assessments of subclinical hypothyroidism typically focus on hormone levels, recent research has revealed a crucial role for the thyroid-stimulating hormone receptor (TSHR) in cardiovascular risk. TSHR belongs to the glycoprotein hormone receptor (GPHR) family, which is a subgroup of class A G protein-coupled receptors (GPCRs). While TSHR is predominantly expressed in thyroid follicular cells, it has also been identified in extra-thyroidal tissues such as the heart and vascular endothelium, indicating a direct role in cardiovascular function and pathology.^3^ Elevated TSH levels in SCH may induce aberrant TSHR gene expression in cardiovascular tissues, leading to inflammation, oxidative stress, and endothelial dysfunction — key mechanisms in atherosclerosis and heart failure.

 Thyroid-stimulating hormone receptor (TSHR) is traditionally recognized for its role in thyroid function; however, emerging evidence indicates its expression in various extra-thyroidal tissues, including the heart, adipose tissue, and skin.^4^ Notably, studies have demonstrated that TSHR expression is upregulated during adipocyte differentiation, suggesting a potential role in metabolic processes.^5^ While these findings implicate TSHR in peripheral tissues, the direct association between TSHR gene expression and cardiovascular risk remains underexplored. Previous research has primarily focused on TSHR mutations and their effects on thyroid disorders, with limited investigation into the receptor’s extra-thyroidal expression and its implications for cardiovascular health. For instance, studies have linked TSHR mutations to thyroid diseases but have not extensively examined their impact on cardiovascular outcomes.^6^ Our study aims to bridge this gap by evaluating TSHR gene expression in cardiovascular blood samples and assessing its association with cardiovascular risk markers in the study population, thereby providing novel insights into the extra-thyroidal role of TSHR in cardiovascular pathophysiology.

 Systemic molecular markers extend beyond receptor activity, providing critical insights into the pathological landscape of subclinical hypothyroidism (SCH). Among these, pro-inflammatory cytokines, especially interleukin-6 (IL-6), have been linked to endothelial dysfunction, plaque vulnerability, and increased cardiovascular risk. Elevated IL-6 levels in SCH patients correlate strongly with early atherosclerotic changes and impaired cardiac function.^7^ In parallel, oxidative stress markers such as 3-nitrotyrosine serve as indicators of nitrosative damage caused by reactive nitrogen species, contributing significantly to myocardial injury and vascular remodeling.^8^ Collectively, these markers reflect a chronic state of low-grade inflammation and oxidative stress, which likely contributesto the cardiovascular complications associated with SCH.

 Understanding these molecular markers is essential to revealing the often-hidden cardiovascular damage present in subclinical hypothyroidism. By evaluating systemic inflammation, oxidative stress, and TSH receptor (TSHR) gene expression together, we gain a more comprehensive view of cardiovascular risk that goes beyond traditional thyroid hormone measurements. This study aims to investigate how TSHR gene expression, in conjunction with systemic markers such as TSH, IL-6, and 3-nitrotyrosine, contributes to the development of cardiovascular complications in individuals with subclinical hypothyroidism. Focusing on these molecular factors deepens our understanding of SCH-related cardiovascular disease and paves the way for targeted therapies in this often-overlooked group.

## Materials and Methods

###  Study Design

 This case-control study was conducted to evaluate the cardiovascular risks and underlying vulnerabilities associated with subclinical hypothyroidism, with the aim of enhancing the understanding of its impact on cardiovascular health and informing early intervention strategies. This study focused on evaluating biochemical and molecular markers associated with cardiovascular risk. The study adhered to the ethical principles outlined in the Declaration of Helsinki (1964) and its subsequent revisions. Ethical approval was obtained from the Institutional Ethics Committee of Genetika (IECG) under Ref. No: 15/2022/IECG.

###  Study Area and Population

 A total of 300 participants between the ages of 20 and 60 were recruited for the study, including 150 individuals diagnosed with subclinical hypothyroidism (case group) and 150 age- and sex-matched healthy individuals (control group). Cases were defined as adults with subclinical hypothyroidism (elevated TSH 4.5–10 mIU/L with normal free T4) without any established cardiovascular disease, as confirmed through their medical records. Participants were selected from multiple hospitals in Thiruvananthapuram and nearby areas, while all laboratory investigations were carried out at Genetika, Centre for Advanced Genetic Studies, Thiruvananthapuram.

###  Inclusion Criteria

- Individuals aged 20-60 years.
- Participants diagnosed with subclinical hypothyroidism, defined by elevated TSH levels (4.5–10 mIU/L) and normal free T4.
- Healthy individuals with normal thyroid function (TSH and free T4 levels) for the control group.
- Participants without established cardiovascular disease or metabolic disorders.
- Willing to participate and provide informed written consent

###  Exclusion Criteria

- Individuals younger than 20 years or older than 60 years.
- Individuals with thyroid dysfunction (TSH > 10 mIU/L or abnormal free T3 or free T4 levels).
- Pregnant or lactating women.
- Current use of thyroid medications or lipid-lowering drugs.
- Participants with any known cardiovascular disease (e.g., coronary artery disease, heart failure, arrhythmias) or metabolic disorders.
- Any other chronic illnesses or conditions that could interfere with study outcomes.

###  Data Gathering

 A comprehensive set of demographics, clinical, and biochemical data was collected through personal interviews using a structured questionnaire, including details on sociodemographic information, physical metrics, lifestyle habits, and family history of diabetes. Written informed consent was obtained from all participants prior to enrollment.

###  Sample Collection and Preparation

 Fasting venous blood (6–8 mL) was collected from each participant under standard aseptic conditions and distributed into EDTA and plain tubes. Serum was separated from plain tubes via centrifugation for the analysis of hormonal, inflammatory, and oxidative stress markers. EDTA samples were used for molecular procedures, including RNA extraction, cDNA synthesis, and Real-Time PCR to evaluate TSHR gene expression.

###  Laboratory Investigations

 Blood samples were processed for biochemical and hormonal analysis using standardized laboratory protocols. The inflammatory marker IL-6 and the oxidative stress marker 3-nitrotyrosine were quantified using enzyme-linked immunosorbent assay (ELISA) kits procured from Origin Diagnostics & Research, Kerala, India, which report acceptable intra- and inter-assay variability. IL-6 and 3-nitrotyrosine were selected as systemic biomarkers to specifically assess inflammatory and nitrosative-stress pathways implicated in endothelial dysfunction and cardiovascular disease. Although other well-established CVD biomarkers such as hs-CRP, TNF-α, and oxidized LDL are widely used, the study focused on mechanistic insights into inflammation–nitrosative stress interactions; therefore, these markers were prioritized. The hormonal marker TSH was quantified using the chemiluminescent immunoassay (CLIA) method. All assays were conducted strictly according to the manufacturer’s guidelines to maintain accuracy and reproducibility of the results.

###  Genetic Analysis

####  RNA isolation and cDNA synthesis 

 RNA was isolated using an RNA extraction kit, quantified with a biospectrometer, and then reverse transcribed into cDNA using a cDNA synthesis kit.

####  Real-Time PCR (RT-PCR)

 TSHR gene expression was analyzed using RT-PCR with specific primers (Forward: 5’-CTGCTCTCATTACACATCAAGGAC-3’ and Reverse: 5’-GTGACACACAGACTATGCTTC-3’) and GAPDH as the reference housekeeping gene. The RT-PCR was performed on a CFX Opus 96 Real-Time PCR system. A 20 μL PCR reaction mixture was prepared, consisting of 2X Real-Time PCR Master Mix, primers, cDNA, and nuclease-free water. The thermal cycling conditions included an initial pre-denaturation at 95°C for 5 minutes, followed by 30-40 cycles of denaturation at 94°C for 1 minute, annealing at 56°C for 1 minute, and extension at 72°C for 1 minute. A final extension at 72°C for 10 minutes was performed, followed by melt curve analysis. Gene expression levels were normalized to the housekeeping gene, and relative expression was calculated using the (2^−ΔΔCt^) method.

## Results

 Statistical analyses included normality testing, group comparisons (t-test, Mann–Whitney U, chi-square), Spearman’s correlation, and ROC curve analysis (markers with AUC ≥ 0.65 were included in the models). Firth’s logistic regression identified predictors of case status, with gene expression classified as dysregulated or normal. Multiple linear regression with robust errors examined factors influencing gene expression. Analyses were performed using Stata 17.0.

Table 1 presents the normality assessment of biochemical, hormonal, and molecular variables in cases and controls. A *P *value < 0.05 indicates non-normal distribution. Most variables showed deviation from normality in at least one group. TSH levels were non-normally distributed, especially among cases. Interleukin-6 was normally distributed in cases but not in controls, while 3-nitrotyrosine showed non-normality in both groups. TSH receptor gene expression was non-normal in cases but normal in controls. These results support the use of nonparametric tests for further analysis.

**Table 1:** Shapiro-Wilk Normality Test Outcomes for Cases and Controls

**Variable**	**Cases**	**Controls**
Thyroid Stimulating Hormone (TSH)	*P* < 0.01	*P* < 0.01
Inflammatory Marker (Interleukin-6)	0.05376	*P* < 0.01
Oxidative Stress Marker (3-Nitrotyrosine)	*P* < 0.01	*P* < 0.01
TSH Receptor Gene Expression (2^-ΔΔCT^)	*P* < 0.01	0.12116

The table presents p-values indicating the statistical significance of differences in key biomarkers between subclinical hypothyroidism cases and controls. Significant elevations were observed in TSH, 3-nitrotyrosine, and TSH receptor gene expression among cases. Interleukin-6 exhibited a borderline association in cases and a significant association in controls, whereas TSH receptor gene expression (2^-ΔΔCT) did not show a significant difference in controls. **TSH:** Thyroid-Stimulating Hormone; **IL-6:** Interleukin-6; **2^-ΔΔCT:** Fold change in gene expression*. P < 0.01 considered statistically significant.*

 The descriptive statistics of key biochemical parameters are summarized in Table 2. Compared to controls, cases exhibited significantly higher levels of TSH, IL-6, and nitrotyrosine, alongside increased TSH receptor gene expression (all *P* < 0.01). These findings collectively indicate heightened hormonal, inflammatory, and oxidative stress, as well as altered molecular activity in cases.

**Table 2:** Descriptive Statistics of the Biochemical Parameters for cases and controls

**Variable Name**	**Cases (Min - Max)**	**Cases (Mean±SD)**	**Controls (Min - Max)**	**Controls (Mean±SD)**	***P *** **value**
Thyroid Stimulating Hormone (TSH, mIU/L)	6.17 – 20.14	11.0 ± 3.1	0.42 – 4.70	2.2 ± 0.9	*P* < 0.01
Interleukin-6 (pg/mL)	4.25 – 18.6	10.6 ± 2.7	0.50 – 7.14	3.1 ± 1.8	*P* < 0.01
Nitrotyrosine (ng/mL)	7.62 – 89.6	33.9 ± 17.4	6.32 – 33.7	17.5 ± 5.6	*P* < 0.01
TSH Receptor Gene Expression (2^-ΔΔCT^)	0.69 – 3.54	2.0 ± 0.8	0.53 – 1.42	1.0 ± 0.1	*P* < 0.01

The table presents the minimum, maximum, mean, and standard deviation (SD) values of key biochemical and molecular markers in subclinical hypothyroidism cases and controls. Statistically significant differences (*P* < 0.01) were observed for all parameters, including TSH, Interleukin-6, 3-nitrotyrosine, and TSH receptor gene expression (2^-ΔΔCT^), indicating elevated hormonal, inflammatory, oxidative stress, and gene expression levels among cases. TSH: Thyroid-Stimulating Hormone; IL-6: Interleukin-6; 2^-ΔΔCT^: Fold change in gene expression; SD: Standard Deviation; P < 0.01 considered statistically significant

**Figure 1:**
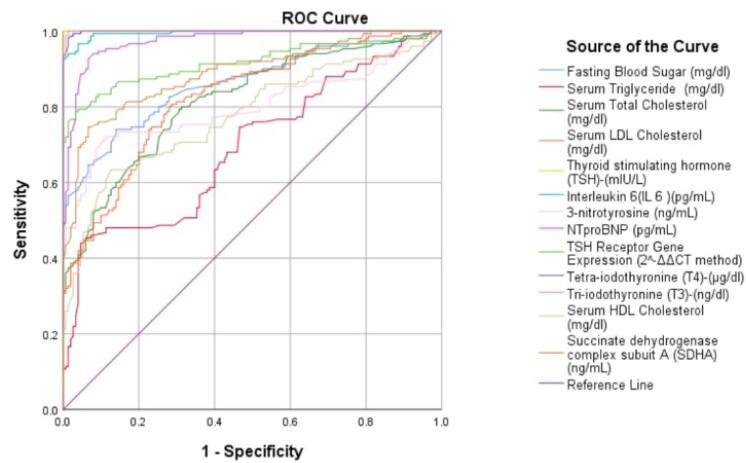
Diagnostic Performance of Biomarkers

 The ROC (Receiver Operating Characteristic) curve in Figure 1 illustrates the diagnostic performance of key biomarkers in distinguishing between the two groups (n = 300). Among metabolic indices, fasting blood sugar showed good discrimination (AUC = 0.86, 95% CI 0.81–0.91), while LDL-C (AUC = 0.83, 95% CI 0.77–0.88) and total cholesterol (AUC = 0.82, 95% CI 0.76–0.87) also displayed strong predictive ability. Triglycerides (AUC = 0.69, 95% CI 0.62–0.76) and HDL-C (AUC = 0.78, 95% CI 0.71–0.84) exhibited moderate accuracy. Thyroid hormones performed poorly, with T3 (AUC = 0.53, 95% CI 0.46–0.59) and T4 (AUC = 0.55, 95% CI 0.48–0.62) failing to reach significance. In contrast, inflammatory and oxidative stress markers displayed excellent discriminative capacity: IL-6 (AUC = 0.99, 95% CI 0.98–1.00) and NT-proBNP (AUC = 0.97, 95% CI 0.95–0.99) were the most powerful predictors, followed by SDHA (AUC = 0.89, 95% CI 0.84–0.94) and 3-nitrotyrosine (AUC = 0.80, 95% CI 0.73–0.86). TSHR gene expression showed a strong discriminative ability (AUC = 0.91, 95% CI 0.86–0.95), indicating its diagnostic potential in identifying thyroid dysregulation linked to systemic inflammation and oxidative stress. TSH showed perfect separation between groups (AUC = 1.00, 95% CI 0.99–1.00), likely reflecting strong disease association but requiring validation in larger cohorts.

**Figure 2:**
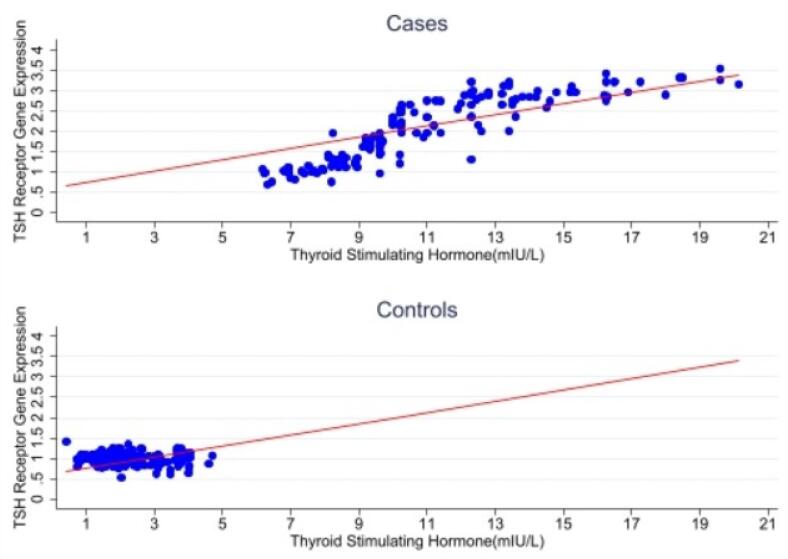
Scatter Plot Analysis: Thyroid Stimulating Hormone (TSH) and TSHR Gene Expression in Cases and Controls

 In cases (n = 150), a strong positive correlation is observed (r = 0.81, < 0.01), indicating upregulation of TSHR with rising TSH, consistent with thyroid dysregulation. In controls (n = 150), the trend is weak and nearly flat (r = 0.32, = 0.07), with low variability, suggesting well-regulated thyroid function (Figure 2).

**Figure 3:**
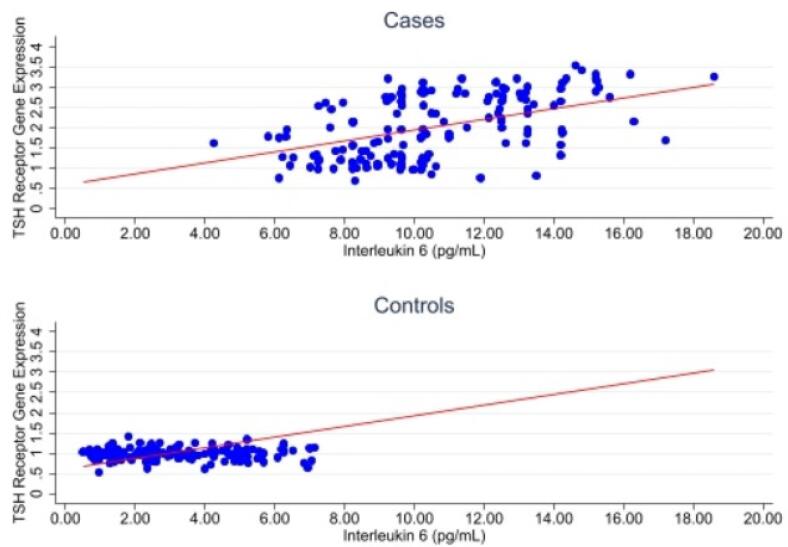
Scatter Plot Analysis: Interleukin 6 and TSH Receptor Gene Expression

 In cases (n = 150), a moderate positive correlation is observed (r = 0.61, < 0.01), suggesting a potential link between inflammation and thyroid receptor regulation. In controls (n = 150), a weaker, non-significant trend is noted (r = 0.27, = 0.10), with lower IL-6 levels and limited TSHR gene expression variability (Figure 3).

**Figure 4:**
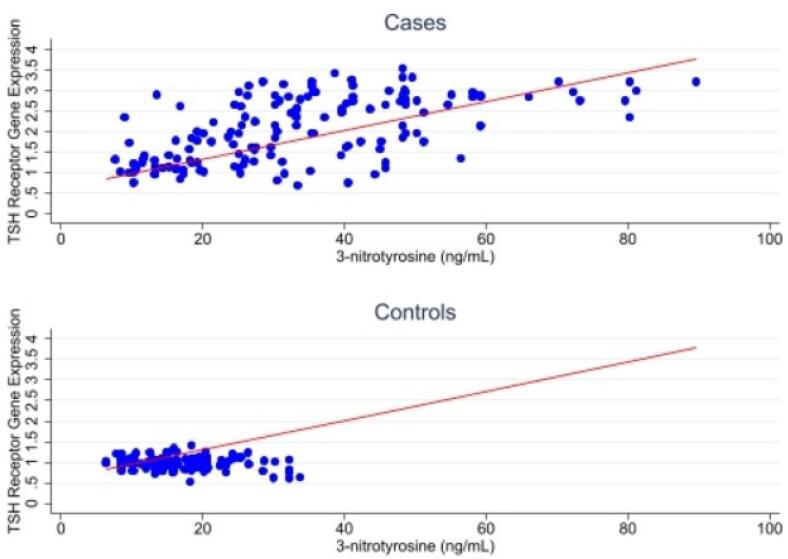
Scatter Plot Analysis: 3-nitrotyrosine and TSH Receptor Gene Expression

 In cases (n = 150), a moderate positive correlation is observed (r = 0.58, < 0.01), suggesting a link between oxidative stress and thyroid receptor activity. In controls (n = 150), a weaker and non-significant correlation is noted (r = 0.29, *P* = 0.09), with narrower variability in both 3-nitrotyrosine levels and TSHR gene expression (Figure 4).

**Figure 5:**
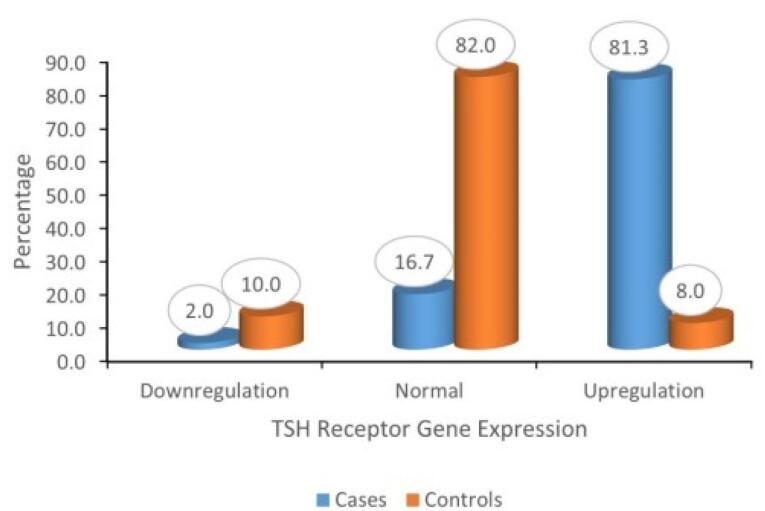
Distribution of TSH Receptor Gene Expression

 As shown in Figure 5, Upregulation was observed in 81.3% of cases versus 8.0% of controls, while normal expression predominated in controls (82.0%) but was less common in cases (16.7%). Downregulation was rare in both groups. The difference between groups was statistically significant (χ^2^ = 163.19, *P* < 0.001), supporting the role of TSHR dysregulation in subclinical hypothyroidism and its potential link to cardiovascular risk.

 Firth logistic regression analysis revealed that dysregulated TSH receptor gene expression was strongly associated with case status in the unadjusted model but lost significance after adjustment, indicating possible confounding. Elevated TSH levels, however, remained a significant independent predictor of case status (OR = 6.14, *P* < 0.05), emphasizing the key role of hormonal imbalance. Nitrotyrosine showed a non-significant trend, suggesting a limited independent effect. These findings are detailed in Table 3.

**Table 3:** Association of TSH Receptor Gene Dysregulation and Biochemical Markers with Case-Control Status Using Firth Logistic Regression

**Variables**	**Model 1 OR [95% CI]**	**Model 2 OR [95% CI]**
TSH Receptor Gene Dysregulation	22.08 [12.22 – 40.01]**	2.43 [0.06 – 105.11]
TSH (µIU/mL)	–	6.14 [1.15 – 32.81]*
Nitrotyrosine (ng/mL)	–	0.88 [0.72 – 1.08]

*Significant at *P* < 0.05, **Significant at *P* < 0.01 The table presents results from logistic regression models assessing predictors of subclinical hypothyroidism. Model 1 shows unadjusted odds ratios (OR) with 95% confidence intervals (CI), while Model 2 includes adjusted estimates. TSH receptor gene dysregulation demonstrated a strong unadjusted association, but the effect diminished after adjustment. TSH remained a significant independent predictor in the adjusted model. Nitrotyrosine showed no significant association. **OR:** Odds Ratio; **CI:** Confidence Interval; **TSH:** Thyroid-Stimulating Hormone; **µIU/mL:** Micro-International Units per Milliliter; **ng/mL:** Nanograms per Milliliter. *P < 0.05; *P< 0.001

 Adjusted regression models included the following covariates: age, BMI, smoking status, alcohol use, comorbidities (hypertension, diabetes, dyslipidemia), and relevant biochemical parameters (fasting glucose, lipid profile, and serum TSH levels) to assess independent associations. Table 4 shows that 3-nitrotyrosine was significantly positively associated with gene expression in cases (*P* < 0.001), suggesting that oxidative stress may drive upregulation. In controls, the association was not significant but showed a negative trend (*P* = 0.075). IL-6 had no significant effect in either group.

**Table 4:** Multiple Linear Regression Estimates for Predictors of TSH Receptor Gene Expression Among Cases and Controls

**Variable**	**Model 1 Coef. [95% CI]**	**Model 2 Coef. [95% CI]**
IL-6 (pg/mL)	–0.0020 [–0.0268 – 0.0229]	0.0134 [–0.0026 – 0.0293]
3-Nitrotyrosine (ng/mL)	0.0065 [0.0033 – 0.0096]***	–0.0042 [–0.0089 – 0.0004]
Constant	0.9476 [–0.0759 – 1.9711]	1.2667 [0.8254 – 1.7080]***

***Significant at *P* < 0.001 The table presents coefficients (Coef.) and 95% confidence intervals (CI) from linear regression models evaluating the effect of inflammatory and oxidative stress markers on TSH receptor gene expression. Model 1 reflects unadjusted associations, while Model 2 is adjusted for relevant covariates. In cases, 3-nitrotyrosine showed a significant positive association with gene expression in the unadjusted model (*P* < 0.001), but the association reversed and lost significance after adjustment. IL-6 showed no significant association in either model. **IL-6:** Interleukin-6; **Coef.:** Regression Coefficient; **CI:** Confidence Interval; **pg/mL:** Picograms per Milliliter; **ng/mL:** Nanograms per Milliliter. **P < 0.001

## Discussion

 Subclinical hypothyroidism (SCH), defined by elevated serum thyroid-stimulating hormone (TSH) levels with normal circulating thyroid hormones, has increasingly been recognized as a potential risk factor for cardiovascular disease (CVD). While hormonal assessments are routinely used in SCH diagnosis and management, emerging research suggests that molecular and systemic biomarkers may offer deeper insights into the pathophysiological mechanisms linking thyroid dysfunction to cardiovascular risk. Among these, the thyroid-stimulating hormone receptor (TSHR) gene has drawn attention for its potential regulatory roles beyond the thyroid gland.

 A review of existing literature revealed that most studies on TSHR gene abnormalities have focused primarily on genetic mutations, particularly in the context of hypothyroidism or pediatric thyroid disorders.^9-11^ Additionally, a study by Lu et al highlighted the extrathyroidal expression of TSHR, particularly in adipose tissue, and its influence on adipocyte differentiation and obesity, further suggesting a broader systemic role for TSHR.^5^ However, there is a distinct lack of studies examining the direct expression of the TSHR gene in adults with subclinical hypothyroidism and its potential association with cardiovascular risk.

 To address this gap, the present study aimed to investigate the expression of the TSHR gene in individuals with subclinical hypothyroidism and evaluate its relationship with cardiovascular risk by examining relevant biochemical, hormonal, inflammatory, and oxidative stress markers. This approach seeks to move beyond traditional hormonal evaluations to uncover molecular predictors that may better inform the early identification and management of cardiovascular risk in SCH subjects.

 Our findings demonstrated a significant upregulation of TSHR gene expression in SCH subjects compared to healthy controls (Table 2), with 81.3% of cases showing upregulation versus only 8.0% of controls (Figure 5). Elevated TSH levels were significantly correlated with increased TSHR expression, particularly in the case group (Figure 2), highlighting a potential feedback mechanism or compensatory upregulation in response to persistent TSH elevation. This is consistent with earlier reports indicating that TSH can modulate the expression of its receptor in a dose-dependent manner.^12^ However, while most previous studies focused on mutations^9^ or pediatric populations ^10,11^, our study extends these findings to adult SCH subjects and suggests a functional role of TSHR gene activity beyond the thyroid, particularly in the context of cardiovascular risk.

 In addition to hormonal markers, the study evaluated inflammatory and oxidative stress biomarkers. Interleukin-6 (IL-6), a pro-inflammatory cytokine associated with endothelial dysfunction and atherogenesis, was significantly elevated in SCH cases (Table 2) and showed a mild positive correlation with TSHR gene expression (Figure 3). Though this association was not statistically significant in the regression model (Table 4), it may still suggest an inflammatory influence on TSHR regulation. Prior studies have reported elevated IL-6 in SCH, potentially linking thyroid dysfunction to systemic inflammation and cardiovascular events.^13^ In the present study, IL-6 levels were significantly higher among cases compared to controls, indicating a heightened inflammatory response associated with disease status. The magnitude of IL-6 elevation observed is comparable to levels reported in patients with early cardiovascular and endocrine inflammatory conditions, suggesting that a similar low-grade systemic inflammation may underlie the observed pathology.

 Oxidative stress, as measured by 3-nitrotyrosine, was also significantly higher in cases than controls (Table 2) and demonstrated a significant positive association with TSHR expression in cases (Table 4), suggesting oxidative stress may be a driver of TSHR upregulation. This association was also supported visually in Figure 4. Although MDA and 8-OHdG are widely used oxidative stress markers, we selected 3-nitrotyrosine to provide a more novel mechanistic insight. Unlike MDA (lipid peroxidation) and 8-OHdG (DNA oxidation), 3-nitrotyrosine specifically reflects nitro-oxidative stress and protein nitration, offering a more direct indication of oxidative modification at the protein level. These findings support the hypothesis that oxidative stress plays a regulatory role in thyroid receptor gene expression, consistent with previous literature linking oxidative stress to both thyroid dysfunction and cardiovascular pathology.^14^

 Although TSHR gene expression showed strong associations in unadjusted analyses, its predictive power was attenuated after adjustment for potential confounders (Table 3, Model 1). This indicates that TSHR may not serve as an independent predictor in the studied population. Consequently, while TSHR remains of interest mechanistically, its utility as a standalone diagnostic biomarker should be interpreted cautiously. Future studies with larger cohorts and comprehensive multivariate analyses are warranted to clarify its independent predictive value. Notably, elevated TSH remained a significant independent predictor of case status (OR = 6.14, *P* < 0.05), reaffirming its role in the pathogenesis of SCH and associated cardiovascular alterations. This finding aligns with large-scale epidemiological studies reporting increased cardiovascular morbidity and mortality in SCH patients with higher TSH levels.^15^ In our study, although the association between TSHR dysregulation and clinical outcomes lost statistical significance after adjustment, the persistence of a relationship between oxidative stress markers and TSHR expression suggests that TSH may indeed act as a mediator. Oxidative stress is known to disrupt hypothalamic–pituitary–thyroid (HPT) axis homeostasis, leading to alterations in TSH secretion. Elevated or dysregulated TSH could, in turn, influence TSHR gene expression, given that receptor expression is responsive to ligand availability and signalling feedback. Therefore, one plausible explanation is that oxidative stress triggers changes in TSH secretion, and this altered TSH milieu secondarily modulates TSHR expression.

 While our data cannot establish causality, this hypothesis highlights an axis in which oxidative stress, TSH levels, and TSHR expression are interconnected. Future studies employing mediation analysis, longitudinal follow-up, and functional assays could help disentangle this relationship and clarify whether TSH functions as a true mediator or whether oxidative stress exerts parallel but independent effects on both TSH and TSHR expression.

 Scatter plot analyses (Figures 2–4) illustrated strong positive correlations between TSH, IL-6, and 3-nitrotyrosine with TSHR gene expression in SCH cases, while these associations were minimal or absent in the control group. These patterns underscore the altered biochemical and molecular environment in SCH, where hormonal imbalance, inflammation, and oxidative stress converge to influence TSHR gene activity.

 Lastly, the ROC curve analysis (Figure 1) confirmed the excellent diagnostic performance of TSH, IL-6, 3-nitrotyrosine, and TSHR gene expression, with AUC values ≥ 0.65, supporting their utility in distinguishing SCH cases at cardiovascular risk from healthy individuals.

 This study provides novel insights into TSH receptor (TSHR) gene expression in adults with subclinical hypothyroidism (SCH) and its potential link to cardiovascular disease (CVD), a topic that has been largely underexplored. The inclusion of well-defined case (SCH) and control (healthy) groups, matched in number and recruited from the same region, helps reduce selection bias and allows for meaningful comparison. The study further benefits from its comprehensive biomarker profiling, incorporating hormonal (TSH), inflammatory (IL-6), oxidative stress (3-nitrotyrosine), and TSHR gene expression. This integrative approach provides a more holistic understanding of the biochemical and molecular alterations in SCH. In addition, the application of rigorous statistical methods, including Shapiro-Wilk normality tests, Mann–Whitney U, Firth’s logistic regression, and multiple linear regression with robust error estimation, enhances the validity of the findings. The use of ROC curve analysis (Figure 1) to assess the discriminatory power of biomarkers further supports their diagnostic relevance.

 Although MDA and 8-OHdG are widely used oxidative stress markers, we selected 3-nitrotyrosine to provide a more novel mechanistic insight. Unlike MDA (lipid peroxidation) and 8-OHdG (DNA oxidation), 3-nitrotyrosine specifically reflects nitro-oxidative stress and protein nitration, offering a more direct indication of oxidative modification at the protein level.

 Despite these strengths, the study has certain limitations inherent to the case–control design. First, although real-time PCR was used to quantify gene expression, protein-level validation (e.g., Western blot or immunohistochemistry) of TSHR was not conducted, which may limit conclusions about receptor functionality. Our focus was on quantifying gene expression levels, which is appropriately measured using RT-PCR rather than Western blot or immunohistochemistry (IHC). While RT-PCR measures mRNA levels (indicating gene expression at the transcription stage), Western blot and immunohistochemistry (IHC) measure the resulting protein; therefore, they assess protein abundance and localization, not mRNA expression.

 As post-transcriptional mechanisms can influence protein levels independent of mRNA expression, the absence of protein-level validation restricts the ability to determine whether increased mRNA expression results in functional protein upregulation. Thus, conclusions regarding functional TSHR dysregulation should be interpreted cautiously.

 Additionally, this study was conducted at a single center in South India, which may limit the generalizability of results to other ethnic or geographic populations. Although cardiovascular risk markers were assessed in SCH participants, none of the participants had diagnosed cardiovascular disease, and no direct measurements of cardiovascular structure or function were performed. Including such assessments in future research may provide a more comprehensive understanding of cardiac involvement. Furthermore, because the case–control design is observational, causality cannot be established. The associations observed should therefore be interpreted with caution and warrant confirmation in longitudinal or interventional studies.

## Conclusion

 The study demonstrates that individuals with subclinical hypothyroidism exhibit significantly elevated TSH, IL-6, and 3-nitrotyrosine levels, as well as TSH receptor gene expression compared to healthy controls. ROC analysis confirmed the strong discriminatory power of these markers, especially TSH and TSHR expression. While TSHR gene dysregulation was strongly associated with disease status in unadjusted models, TSH remained the most consistent independent predictor after adjustment. Oxidative stress, as indicated by 3-nitrotyrosine, showed a significant positive association with TSHR expression among cases, highlighting its potential regulatory role. These findings underscore the interplay between hormonal, inflammatory, and oxidative stress pathways in SCH and highlight associative relationships rather than predictive associations for cardiovascular risk. However, since no direct cardiovascular clinical or structural outcomes (e.g., echocardiography, carotid intima–media thickness, myocardial infarction, or stroke) were assessed, the results should be interpreted as associative rather than predictive, warranting further longitudinal or interventional studies to evaluate causal links.

## Competing Interests

 The authors declare that they have no competing interests.

## Ethical Approval

 Ethical approval (15/2022/IECG) was secured from the Institutional Ethics Committee of Genetika.
